# The L-shaped selection algorithm for multitrait genomic selection

**DOI:** 10.1093/genetics/iyac069

**Published:** 2022-04-28

**Authors:** Fatemeh Amini, Guiping Hu, Lizhi Wang, Ruoyu Wu

**Affiliations:** Department of Industrial and Manufacturing Systems Engineering, Iowa State University, Ames, IA 50011, USA; Department of Industrial and Manufacturing Systems Engineering, Iowa State University, Ames, IA 50011, USA; Department of Sustainability, Golisano Institute for Sustainability, Rochester Institute of Technology, Rochester, NY 14623, USA; Department of Industrial and Manufacturing Systems Engineering, Iowa State University, Ames, IA 50011, USA; Department of Mathematics, Iowa State University, Ames, IA 50011, USA

**Keywords:** multitrait genomic selection, index selection, L-shaped selection, Pareto optimality, genetic diversity

## Abstract

Selecting for multiple traits as opposed to a single trait has become increasingly important in genomic selection. As one of the most popular approaches to multitrait genomic selection, index selection uses a weighted average of all traits as a single breeding objective. Although intuitive and effective, index selection is not only numerically sensitive but also structurally incapable of finding certain optimal breeding parents. This paper proposes a new selection method for multitrait genomic selection, the L-shaped selection, which addresses the limitations of index selection by normalizing the trait values and using an L-shaped objective function to find optimal breeding parents. This algorithm has been proven to be able to find any Pareto optimal solution with appropriate weights. Two performance metrics have also been defined to quantify multitrait genomic selection algorithms with respect to their ability to accelerate genetic gain and preserve genetic diversity. Computational experiments were conducted to demonstrate the improved performance of L-shaped selection over-index selection.

## Introduction

The effectiveness of genomic selection (GS) in accelerating genetic gain in plant and animal breeding programs (Jannink *et al.* 2010; Meuwissen and Goddard 2010; Desta and Ortiz 2014; Rutkoski *et al.* 2016) has motivated breeders to apply the technique for multiple traits, including yield, quality, and tolerance to biotic and abiotic stresses (Jia and Jannink 2012). Breeders are more likely to invest in GS programs that are capable of improving multiple traits of a crop rather than a single trait throughout the generations (Lynch and Walsh 1998; Bernardo 2002).

Existing approaches for multitrait genomic selection (MTGS) include tandem selection, independent culling selection, and index selection. Tandem selection treats MTGS as the aggregation of multiple single-trait GS programs and selects for the traits sequentially (Burgess and West 1993). Independent culling selection sets a minimum threshold (i.e. culling levels) for each trait and only selects individuals that exceed the culling levels for all traits (Lorenzana and Bernardo 2009). Index selection converts MTGS to a single-trait GS by using a linear combination of individual traits weighted by their importance as the breeding objective (Hazel and Lush 1942; Hazel 1943; Williams 1962). Recently, Moeinizade *et al.* (2020) proposed a new algorithm for MTGS that maximizes one trait subject to the constraints that another trait falls within a desirable range.

Index selection has heretofore been a commonly used approach to MTGS due to its capability to select for multiple traits simultaneously and its flexibility to assign different weights according to the relative importance of the traits. In contrast, tandem selection can only select for one trait at a time, and independent culling selection may eliminate an otherwise high-performing individual due to its minor shortcoming in one trait (Lorenzana and Bernardo 2009). Index selection overcomes such limitations by taking an importance-weighted linear combination of all traits, giving breeders a wide range of trade-off options among the traits to choose from.

However, index selection also suffers from its own limitations, i.e. its numerical sensitivity and inability to find certain optimal selections. In this study, we propose a new approach to MTGS, which uses an L-shaped objective function (as opposed to the linear objective function used in index selection) to select optimal breeding parents that strike a balance among multiple traits with respect to their relative importance. This algorithm not only overcomes the 2 limitations of index selection but also demonstrates superior performance with respect to both accelerating genetic gain and preserving genetic diversity.

The rest of the paper is organized as follows. In *Materials and methods*, we formally formulate MTGS as a multiobjective optimization problem, introduce the L-shaped selection algorithm, and present the mathematical properties that allow it to overcome the limitations of index selection. Moreover, we define 2 metrics for assessing the performances of MTGS algorithms in terms of accelerating genetic gain and preserving genetic diversity. In *Computational experiments*, we describe the computational experiments that we conducted to compare index selection and L-shaped selection methods. Finally, concluding remarks are made and future research directions are discussed in *Conclusion*.

## Materials and methods

Consider a breeding project that starts with an initial population of plant or animal individuals. A number of crosses are made in each generation to produce a new population of progeny in the next generation until a predefined deadline for the project. Suppose there are multiple traits that the breeders aim to improve through the breeding process. Under the following 3 simplifying assumptions, the focus of our study is to select the right individuals to make the right crosses in order to optimize all traits at the end of the breeding project.

**Assumption 1** There are adequate and reliable genetic data, including genotype and recombination frequencies.

**Assumption 2** All traits are largely determined by additive effects with negligible dominance or epistatic effects.

**Assumption 3** Allele effects for all traits have been estimated sufficiently accurately and constant over the breeding process.

### Problem definition

The objective of MTGS is to select a subset of breeding parents from a group of candidate individuals based on their genotype and estimated allele effects in order to maximize genetic gains with respect to multiple traits over a number of breeding generations. The following nomenclature will be used in this paper.

I set of candidate individuals of plants or animals for selection

J set of loci

K set of traits

$G_{i,j}$ genotype of individual i∈I at locus j∈J

$\beta_{j,k}$ effect of allele j∈J on trait k∈K

$v_{i,k}$ genetic value of individual i∈I on trait k∈K: $v_{i,k}=\sum_{j}G_{i,j}\beta_{j,k}$

*w_k_*weight parameter that indicates the relative importance of trait k∈K

*x_i_* binary variable indicating whether individual i∈I is selected (*x_i_* = 1) or not (*x_i_* = 0)

*S* number of breeding parents to be selected

Without loss of generality, we assume that maximization (rather than minimization) is the direction of improvements for all traits. For a trait *k* that needs to be minimized, we can replace $v_{i,k}$ with $-v_{i,k}$ for all i∈I in a maximization model, which is equivalent to minimizing trait *k*. For traits whose values need to be contained within a desirable range, we can maximize the percentage of individuals in a population whose trait values fall within such a range.

With the above definitions and assumptions, the MTGS can be formulated as the following multiobjective optimization model:

(1) $$\underset{x}{\max}\;\sum_{i}x_{i}v_{i,k}\forall k\in K$$

(2) $$s.t.\;\sum_{i}x_{i}=S$$

(3) $$x_{i}\in \{0,1\}\;\forall i.$$

Here, the objective function (1) is the maximization of all traits of selected individuals. Constraint (2) requires that exactly *S* individuals be selected. Constraint (3) defines *x_i_* as a binary variable for all i∈I.

In multiobjective optimization, a feasible solution is called Pareto optimal if it is not dominated by any other feasible solution. Solution $\hat{x}$ dominates $\tilde{x}$ if $\hat{x}$ is no worse than $\tilde{x}$ in any trait and better in at least one:

$$\sum_{i}{\hat{x}}_{i}v_{i,k}\geq \sum_{i}{\tilde{x}}_{i}v_{i,k},\forall k\in K\;\text{and}\;\sum_{i}{\hat{x}}_{i}v_{i,k}>\sum_{i}{\tilde{x}}_{i}v_{i,k},\exists k\in K.$$

Moreover, the ultimate goal of solving a multiobjective optimization problem is to find not a single Pareto optimal solution but all Pareto optimal solutions that represent the range of possible trade-offs among different traits, referred to as the Pareto frontier. Solutions on the Pareto frontier are not equivalent; rather, they offer nondominated alternatives for decision-makers to choose from, with each alternative being optimal under certain criteria of trade-off among the multiple objectives.

### Index selection

As a widely used selection approach for MTGS, index selection solves a single objective optimization model that maximizes the weighted average of all traits:

(4) $$\underset{x}{\max}\;\sum_{k}w_{k}\sum_{i}x_{i}v_{i,k}$$

(5) $$s.t.\;\sum_{i}x_{i}=S$$

(6) $$x_{i}\in \{0,1\}\;\forall i.$$

Here, the objective function (4) is the weighted average genetic value of all traits. When different weight parameters are used, index selection essentially searches for the convex efficient frontier, which is the subset of Pareto optimal solutions that are on the convex hull of the feasible region.

The following proposition shows that, when strictly positive weights are used for all traits, the optimal solution to index selection must be Pareto optimal to MTGS.

Proposition 1. *If solution* $\hat{x}$*is optimal to (4)–(6) for some* $w_{k}>0,\forall k\in K$*, then* $\hat{x}$*is Pareto optimal to (1)–(3).*

*Proof.* Suppose $\hat{x}$ is not Pareto optimal to (1)–(3). Then, there exists a solution $\tilde{x}$ that dominates $\hat{x}$:

$$\sum_{i}{\tilde{x}}_{i}v_{i,k}\geq \sum_{i}{\hat{x}}_{i}v_{i,k},\forall k\in K\;\text{and}\;\sum_{i}{\tilde{x}}_{i}v_{i,k}>\sum_{i}{\hat{x}}_{i}v_{i,k},\exists k\in K.$$

Since $w_{k}>0,\forall k\in K$, we have

$$\sum_{k}w_{k}\sum_{i}{\tilde{x}}_{i}v_{i,k}>\sum_{k}w_{k}\sum_{i}{\hat{x}}_{i}v_{i,k}.$$

This contradicts the assumption that $\hat{x}$ is optimal to (4)–(6). Therefore, $\hat{x}$ must be Pareto optimal to (1)–(3). □

### Two limitations of index selection

Despite the effectiveness of index selection in finding Pareto optimal solutions to MTGS, it suffers from 2 major limitations. First, numerical solutions to (4)–(6) may be sensitive to the units being used for the genetic effects of different traits. For example, when we try to maximize both plant height and grain yield with their respective weights, different solutions may result from simply changing the units used to measure the 2 traits from inches and bushels per acre to meters and tonnes per hectare. As a result, it is challenging to determine or interpret the numerical values of the weights for different traits.

The second limitation is the inability to discover all Pareto optimal solutions by using different values of weight parameters *w_k_* due to the convexity of the objective function (4) and the potential nonconvexity of the set of Pareto optimal solutions. To illustrate this point, consider the following example.

**Fig. 1.**
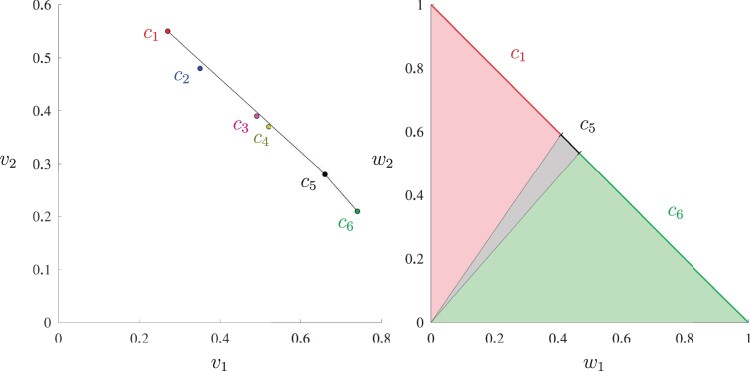
The left subfigure shows the 6 possible crosses in the *v*_1_-*v*_2_ space and the efficient frontier that index selection is able to find. The right subfigure shows that only *c*_1_, *c*_5_, and *c*_6_ could be found to be Pareto optimal using index selection with different weights *w*_1_ and *w*_2_.

Example 1. *Suppose* *2* *individuals are to be selected from* *4* *to make a cross, then there are* *6* *candidate crosses. The genetic values of the* *4* *individuals and* *6* *crosses for* *2* *important traits are summarized in**Tables 1* and 2. *All* *6* *crosses are Pareto optimal since none of them is dominated by another. However, as shown in Fig. 1, the index selection model (4)–(6) can only find* *3* *crosses (c_1_, c_5_, and c_6_) that are on the efficient frontier of the* *6* *crosses in the v_1_-v_2_ space. The other* *3* *will never be selected no matter what non-negative weights w_1_ and w_2_ are used because they are dominated by the line segment between c_1_ and c_5_.*

**Table 1.** Genetic values of 2 traits for 4 individuals.

Individual	$v_{i,k=1}$	$v_{i,k=2}$
*i*_1_	0.05	0.33
*i*_2_	0.22	0.22
*i*_3_	0.30	0.15
*i*_4_	0.44	0.06

**Table 2.** Genetic values of 2 traits for 6 crosses.

Cross	Individuals	$\sum_{i\in c}v_{i,k=1}$	$\sum_{i\in c}v_{i,k=2}$
*c*_1_	(*i*_1_, *i*_2_)	0.27	0.55
*c*_2_	(*i*_1_, *i*_3_)	0.35	0.48
*c*_3_	(*i*_1_, *i*_4_)	0.49	0.39
*c*_4_	(*i*_2_, *i*_3_)	0.52	0.37
*c*_5_	(*i*_2_, *i*_4_)	0.66	0.28
*c*_6_	(*i*_3_, *i*_4_)	0.74	0.21

### L-shaped selection

The proposed L-shaped selection for MTGS can be formulated as the following optimization model:

(7) $$\underset{x}{\max}\;\underset{k}{\min}\frac{\sum_{i}x_{i}{\tilde{v}}_{i,k}}{w_{k}}$$

(8) $$s.t.\;\sum_{i}x_{i}=S$$

(9) $$x_{i}\in \{0,1\}\;\forall i.$$

Here, ${\tilde{v}}_{i,k}=\frac{v_{i,k}-{\underline{v}}_{k}}{{\bar{v}}_{k}-{\underline{v}}_{k}}$ is the normalized genetic value that falls within the range of (0, 1), where ${\underline{v}}_{k}$ and ${\bar{v}}_{k}$ are the lower and upper bounds of trait *k*, which can be obtained, respectively, as ${\underline{v}}_{k}=\underset{i}{\min}(v_{i,k}-\epsilon ),\forall i\in \tilde{I}$ and ${\bar{v}}_{k}=\underset{i}{\max}(v_{i,k}+\epsilon ),\forall i\in \tilde{I}$ for a large set of individuals $\tilde{I}$; here *ϵ* is a small positive value to ensure that the normalized genetic value falls within (0, 1) and not on the boundaries.

This new formulation was designed to address the 2 limitations discussed in *Two limitations of index selection*. The normalized genetic effect ${\tilde{v}}_{i,k}$ is independent of the measurement units being used. The application of this well-known normalization technique in MTGS allows the model to strike a balance among multiple traits in more meaningful terms. After normalization, the numerical values of the weights can be interpreted as the relative importance for each percentage point of improvement in the respective traits. For example, if $w_{1}=0.8$ and $w_{2}=0.2$, then a 1% improvement of trait 1 is 4 times as important as a 1% improvement of trait 2, regardless of the measurement units used for the 2 traits. This interpretation of weights allows breeders to make the most appropriate trade-off among different traits. In certain cases, relative monetary values of the traits can be used as the weights; in other cases, such as when the prices of certain traits are volatile or otherwise hard to determine, the weights can also reflect subject emphases that breeders place on different traits.

The following proposition demonstrates how model (7)–(9) addresses the second limitation.

Proposition 2. *If solution* $\hat{x}$*is Pareto optimal to (1)–(3), then there exists* $w_{k}>0,\forall k\in K$*such that* $\hat{x}$*is optimal to (7)–(9).*

*Proof*. We claim that $w_{k}:=\sum_{i}{\hat{x}}_{i}{\tilde{v}}_{i,k}>0,\forall k\in K$ are such that $\hat{x}$ is optimal to (7)–(9). Suppose not, then there exists a solution $\tilde{x}$ such that

$$\underset{k}{\min}\frac{\sum_{i}{\tilde{x}}_{i}{\tilde{v}}_{i,k}}{w_{k}}>\underset{k}{\min}\frac{\sum_{i}{\hat{x}}_{i}{\tilde{v}}_{i,k}}{w_{k}}=1.$$

This means $\sum_{i}{\tilde{x}}_{i}{\tilde{v}}_{i,k}>w_{k}=\sum_{i}{\hat{x}}_{i}{\tilde{v}}_{i,k},\forall k\in K.$ Since $\sum_{i}{\tilde{x}}_{i}=S=\sum_{i}{\hat{x}}_{i}$, we have

$\sum_{i}{\tilde{x}}_{i}v_{i,k}>\sum_{i}{\hat{x}}_{i}v_{i,k},\forall k\in K.$ This contradicts the assumption that $\hat{x}$ is Pareto optimal to (1)–(3). Therefore, $\hat{x}$ must be optimal to (7)–(9) with $w_{k}:=\sum_{i}{\hat{x}}_{i}{\tilde{v}}_{i,k}\geq 0,\forall k\in K$. □

**Fig. 2.**
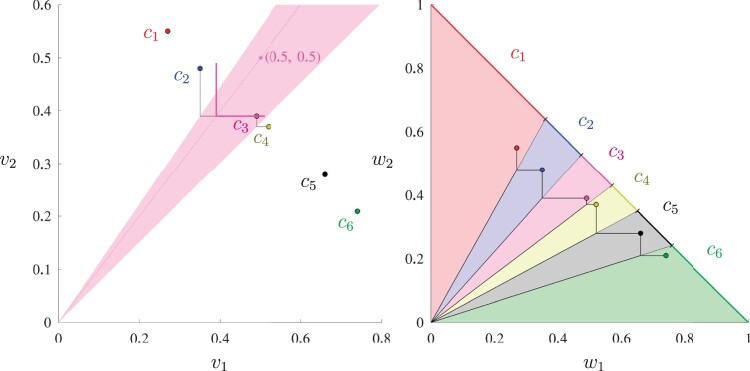
The left subfigure shows the 6 candidate solutions in the *v*_1_-*v*_2_ space and how L-shaped selection uses an L-shaped objective function to search for Pareto optimal solutions. As an example, *c*_3_ is found to be optimal with equal weights on the 2 traits, since it allows the L-shaped objective function to slide the furthest away from the origin toward the direction $\left(v_{1}=w_{1}=0.5,v_{2}=w_{2}=0.5\right)$. Moreover, *c*_3_ is optimal for all weights inside the shaded (unbounded) triangle, the 2 edges of which cross the vertices of 2 L-shaped objective functions with one crossing *c*_2_ and *c*_3_ and the other crossing *c*_3_ and *c*_4_. The right subfigure shows that all 6 candidate solutions can be found to be Pareto optimal using L-shaped selection with different weights *w*_1_ and *w*_2_. The 6 candidate solutions are also plotted in the right subfigure in the *w*_1_-*w*_2_ space to illustrate how different regions of weights are determined.

Proposition 2 is a guarantee that L-shaped selection is able to find any Pareto optimal solution to MTGS with appropriate weight parameters. To illustrate this desirable property, which index selection does not have, we solved Example 1 using L-shaped selection, and results are shown in Fig. 2. The left subfigure illustrates that, in the case of *k *=* *2, model (7)–(9) is trying to slide an L-shaped objective function (hence the name) along the direction from the origin toward (*w*_1_, *w*_2_) to the maximal extent while touching at least one solution with the L-shaped curve. The right subfigure shows the different Pareto optimal solutions that can be found using different combinations of weight parameters *w*_1_ and *w*_2_.

The significance of Proposition 2 is the ability of L-shaped selection to offer a diverse set of alternative breeding solutions on the Pareto frontier that empowers breeders in 3 important ways: (1) understand the set of available trade-offs among multiple traits, (2) realize the outcomes as a result of a wide range of weights being used, and (3) select the most appropriate weights and breeding parents according to the specific breeding objectives and constraints.

### Performance measures of MTGS algorithms

In this section, we define 2 measures, namely Pareto optimality gap and diversity, to evaluate the performance of MTGS algorithms. The motivation is to assess the capability of an algorithm to produce progeny through the breeding process that is not only Pareto optimal but also representative of diverse trade-offs among different traits.

Suppose an MTGS algorithm was used in a number of breeding projects, each with different sets of weight parameters. Let $I^{0}$ denotes the set of individuals produced in the final generation of all breeding projects combined, and let $I^{1}$ denotes a superset of $I^{0}$, possibly also including individuals produced from all other competing algorithms. For any set of individuals $\hat{I}$, we define $P(\hat{I})$ as the Pareto optimal subset of $\hat{I}$:

$$P(\hat{I})=\left\{i:i\in \underset{i\in \hat{I}}{\text{argmax}}\sum_{k\in K}w_{k}v_{i,k},\exists w_{k}>0,\forall k\in K\right\}.$$

The 2 performance measures are defined as follows:

- Pareto optimality gap of $P(I^{0})$ against $P(I^{1})$ is defined as
  $$\sum_{i\in P(I^{0})}\underset{i\prime \in P(I^{1})}{\min}\underset{k\in K}{\min}w_{i,k}{\left(v_{i\prime ,k}-v_{i,k}\right)}_{+},$$

which measures the extent to which set $P(I^{0})$ is dominated by $P(I^{1})$. The term ${\left(v_{i\prime ,k}-v_{i,k}\right)}_{+}:=\max\{v_{i\prime ,k}-v_{i,k},0\}$ detects any positive gap between individuals $i\prime \in P(I^{1})$ and $i\in P(I^{0})$ on trait *k*. Then, the term $\underset{k\in K}{\min}w_{i,k}{\left(v_{i\prime ,k}-v_{i,k}\right)}_{+}$ identifies the smallest weighted gap across all traits in order to determine the extent to which individual *i* is dominated by i′. Next, the term $\underset{i\prime \in P(I^{1})}{\min}\underset{k\in K}{\min}w_{i,k}{\left(v_{i\prime ,k}-v_{i,k}\right)}_{+}$ identifies the individual i′ that dominates *i* by the least amount. This term is the Pareto optimality gap between individual *i* and $P(I^{1})$, and the summation of which over all individuals in $P(I^{0})$ gives the Pareto optimality gap of the set $P(I^{0})$ against $P(I^{1})$.

The Pareto optimality gap can be considered as the minimal amount of trait improvements in $I^{0}$ necessary to break the dominance of $P(I^{1})$ over any individual in $P(I^{0})$. A noteworthy observation here is that the Pareto optimality gap between $i\in P(I^{0})$ and $i\prime \in P(I^{1})$ is zero if $v_{i\prime ,k\prime }=v_{i,k\prime }$ for one trait k′ and $v_{i\prime ,k}>v_{i,k}$ for all others k∈P\k′. This may be counter-intuitive but is also defensible because, although *i* is dominated by i′, it takes an arbitrarily small positive improvement in individual *i* on trait k′ to break the dominance.

- Diversity of $P(I^{0})$ is defined as
  $$\sum_{k\in K}\left[\frac{w_{k}}{\left|P(I^{0})\right|}\sqrt{\sum_{i\in P(I^{0})}\sum_{i\prime \in P(I^{0})}{\left(v_{i,k}-v_{i\prime ,k}\right)}^{2}}\right],$$
  which measures the weighted average Euclidean distance of all Pareto optimal solutions within set $P(I^{0})$. An ideal MTGS algorithm should be able to produce progeny with a small Pareto optimality gap (not being dominated by progeny produced from competing algorithms) and a large diversity (offering different trade-off options in traits).

## Computational experiments

We compared the performances of index selection and L-shaped selection with computational experiments using a maize data set considering 2 traits: plant height and ear diameter.

### Data sets

We used 200 maize inbred lines of 369 shoot apical meristem population distributed across the 10 chromosomes (ISU 2020). We extracted 1,000 single nucleotide polymorphisms (SNPs) out of the total of 1.4 million SNPs that were collected using genome-wide association study used by Leiboff *et al.* (2015), merged with additional SNPs genotyped using tGBS (Schnable *et al.* 2013), and those which were phased using Beagle (Browning and Browning 2009). Recombination rates in this population were estimated using the genetic map developed from the maize nested association mapping population. Genetic effects of the 2 traits, plant height and ear diameter, were extracted from Bernardo and Yu (2007). Both traits are affected by 1,000 loci with pleiotropic effects of varying magnitudes. The genetic correlation of the 2 traits is 0.375. Like index selection, L-shaped selection also applies to both correlated and uncorrelated traits. A similar data set was used in Amini *et al.* (2021), which also showed figures of summary statistics of the data.

### Breeding process

In each simulation of the breeding process, 200 individuals were randomly selected from the 369 inbred lines to form an initial population. In each of the subsequent generations, 2 individuals were selected using either index selection or L-shaped selection to produce 200 progeny in the next generation. The genetic values $v_{i,k}$ of all 200 individuals *i* for the 2 traits *k* in the fifth generation were used for performance analyses. Nine groups of this breeding process were simulated, each for a different set of weight parameters $w_{1}\in \{0.1,0.2,\ldots ,0.9\}$ and $w_{2}=1-w_{1}$ with 10 independent repetitions.

## Results and discussions

**Fig. 3.**
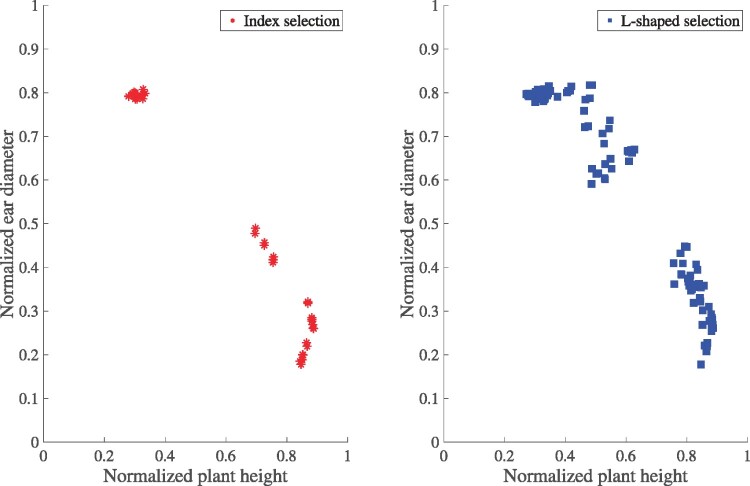
Performance of progeny in the final generation for index selection (left) and L-shaped selection (right).

We compared the performances of index selection and L-shaped selection with respect to normalized plant heights and ear diameters of progeny in the final generation (*T *=* *5), with both traits to be maximized. Figure 3 shows the aggregated results of 90 experiments (9 weights by 10 repetitions) using 2 algorithms; only those progeny that were Pareto optimal within each experiment were plotted. The *x*-axis and *y*-axis represent the normalized plant height and normalized ear diameter, respectively. It can be seen that L-shaped selection resulted in better-performing progeny in terms of both genetic gain and diversity. In fact, the same number of progeny was produced using the 2 selection methods. The reason that the left subfigure had much fewer points than the right one was because, compared with L-shaped selection, much more progeny from index selection were either dominated by or overlapping with other progeny in the same generation.

**Fig. 4.**
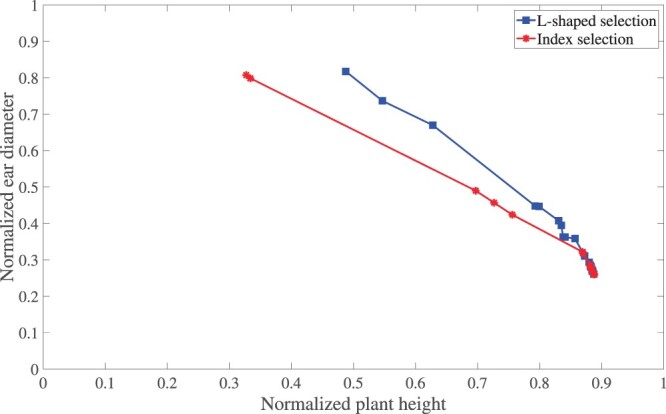
Pareto frontiers of progeny in the final generation for index selection and L-shaped selection.

The optimal Pareto frontier of Fig. 3 is shown in Fig. 4. It can be seen that, for all different sets of weight parameters $w_{1}\in \{0.1,0.2,\ldots ,0.9\}$ and $w_{2}=1-w_{1}$, L-shaped selection outperforms index selection, since the optimal Pareto frontier for L-shaped selection not only dominates that for index selection but also is more diverse.

Table 3 shows the Pareto optimality gap and diversity of all progeny in the final generation. The Pareto optimality gap assesses the capability of a selection method in resulting in Pareto optimal progeny; the lower the gap, the more Pareto optimal the progeny. We assessed the Pareto optimality gap of index selection and L-shaped selection against the combined sets of progeny from the 2 approaches. As can be seen in Table 3, the Pareto optimality gap for L-shaped selection is zero, meaning that none of its Pareto optimal progeny was dominated by those from index selection. In contrast, index selection had a Pareto optimality gap of 0.2916, meaning that the Pareto optimal progeny from index selection was on average dominated by 0.2916 by those from L-shaped selection. The table also shows that progeny in the final generation produced using L-shaped selection were more diverse than those using index selection, in terms of representing different trade-offs between the 2 traits. These observations were consistent with results from Fig. 3.

**Table 3.** Pareto optimality gap and diversity of index selection and L-shaped selection.

Method Metric	Pareto optimality gap	Diversity
Index selection	0.2916	0.1933
L-shaped selection	0	0.2582

### Conclusion

We presented the L-shaped selection for MTGS and demonstrated its improvements over the commonly used index selection in the capability to produce elite progeny with better genetic traits and higher diversity. Motivated by 2 limitations of index selection, the new approach made 2 major contributions to MTGS. First, L-shaped selection is robust against different measurement units used for multiple traits. Second, L-shaped selection guarantees to find all Pareto optimal solutions with appropriate weight parameters. Moreover, we introduced 2 metrics to quantify the capability of MTGS algorithms in accelerating genetic gain and preserving genetic diversity.

Besides theoretical contributions, L-shaped selection also outperformed index selection in computational experiments using a maize data set. The results demonstrate that L-shaped selection outperforms index selection in producing better-performing and more diverse population, considering plant height and ear diameter as 2 traits. Supported by the theoretical results, L-shaped selection is also applicable to other breeding populations with available genotypic and phenotypic data sets.

This study is not without limitations that could be addressed in follow-up research studies. Firstly, one can integrate the objective function in the L-shaped selection formulation in more sophisticated algorithms for GS (De Beukelaer *et al.* 2017; Goiffon *et al.* 2017; Gorjanc *et al.* 2018; Moeinizade *et al.* 2020) rather than a straightforward truncation selection as used in this study. Secondly, although we have considered only 2 traits in our study in order to better visualize the results in 2D figures, the method applies to an arbitrarily large number of traits. A comprehensive case study with appropriate data from a large number of traits would be of interest for a future research project. Thirdly, L-shaped selection was designed to improve the simple nonpenalized index selection. In contrast, the multitrait look-ahead selection (MT-LAS) algorithm (Moeinizade *et al.* 2020) was designed to specifically address the case where one trait is to be maximized and the other restricted between a lower bound and an upper bound, which was comparable with the penalized index selection (Lopez-Cruz *et al.* 2020). However, MT-LAS is much more computationally intensive and not compatible with other MTGS methods, whereas L-shaped selection can be combined with other MTGS methods by replacing their objective functions with the L-shaped one. Finally, it would be more realistic to consider dominance, epistatic, and environmental effects in calculating genetic trait values of individuals (Amini *et al.* 2021), however, only additive effects have been considered in this study.

## Data availability

The datasets used in the computational experiments were derived from sources in the public domain as described in *Data sets*. Computer codes used for the computational experiment were uploaded to GitHub (2022).
